# Randomized Study of enterade^®^ to Reduce Diarrhea in Patients Receiving High-Dose Chemotherapy and Autologous Hematopoietic Stem Cell Transplantation

**DOI:** 10.31557/APJCP.2021.22.1.301

**Published:** 2021-01

**Authors:** Zachariah De Filipp, Brett Glotzbecker, Laura Luque, Haesook T Kim, Katherine M Mitchell, Samuel N Cheuvront, Robert J Soiffer

**Affiliations:** 1 *Blood and Marrow Transplant Program, Massachusetts General Hospital, Boston, MA, USA. *; 2 *Department of Clinical Oncology, Dana Farber Cancer Institute, Boston, MA. USA. *; 3 *Department of Science & Technology, Entrinsic Bioscience, Norwood, MA, USA.*; 4 *Department of Clinical Research, Dana Farber Cancer Institute, Boston, MA, USA. *

**Keywords:** Amino acids, chemotherapy, diarrhea, melphalan, medical-food

## Abstract

High-dose chemotherapy frequently causes injury to the gastrointestinal mucosa, resulting in diarrhea. The purpose of the current study was to assess the tolerability and efficacy of enterade^®^ in reducing ≥ grade 2 diarrhea (G2D) in association with high-dose melphalan followed by autologous stem cell transplantation (ASCT). We conducted a prospective, double blinded, multi-center trial in which 114 subjects were randomized to receive enterade^®^ or placebo twice daily during the transplant hospitalization. Gastrointestinal toxicities (nausea, vomiting, oral mucositis and dysphagia) resulted in poor study compliance in both arms. Among subjects who were able to complete planned therapy (13%), the incidence of G2D was lower for those receiving enterade^®^ as compared to placebo (16% vs 86%, p<0.03). Twice daily oral administration of enterade^®^ and placebo following high-dose chemotherapy and ASCT was not feasible due to significant gastrointestinal toxicities. Future explorations of enterade^®^ should be conducted in populations capable of reasonable oral intake.

## Introduction

High-dose alkylating agents such as cyclophosphamide, melphalan, busulfan, and the use of total body irradiation are commonly used in conditioning regimens for hematopoietic cell transplantation (Morishita et al., 2016). Melphalan represents the mainstay of conditioning for autologous stem cell transplantation (ASCT) in multiple myeloma (MM) and non-Hodgkin lymphoma (NHL) patients. However, its use can be associated with high rates of gastrointestinal (GI) toxicity, leading to significant nausea, diarrhea, poor oral intake and dehydration (Kiss et al., 2014). As a consequence, approximately 40% of patients experience grade 2 or higher diarrhea following melphalan-containing conditioning regimens (Krishna et al., 2011; Sharma et al., 2013). 

The commercial medical food known as enterade^®^ has been used safely by more than 30,000 patients suffering from GI toxicity symptoms in association with cancer therapies. Retrospective open-label clinical studies using enterade^®^ in patients with all tumor types and cancer therapies report reductions in digestive symptoms (nausea, bloating, cramping, diarrhea) and weight loss (Hendrie et al., 2019; Luque et al., 2020). Composite digestive symptoms scores were improved by 78% in subjects with enterade^®^ (n= 60) versus only 7% in subjects without intervention (n= 15) (Luque et al., 2020). These outcomes correspond with pre-clinical studies. Murine studies have demonstrated improvements in the absorptive capacity of the small intestine by rebuilding the villi and reducing antigenic translocation by tightening the mucosal barrier. These changes were associated with reduced nausea (reduced pica weight), improved body weight maintenance, and extended survival time with the administration of enterade^®^ (Yin et al., 2014; Yin et al., 2016; Yin et al., 2017; Gupta et al., 2020). 

 In the current study, we sought to evaluate the tolerability and efficacy of enterade^®^ in reducing ≥ grade 2 diarrhea in association with high-dose melphalan chemotherapy. 

## Materials and Methods

This was a prospective, double blinded, 2-arm randomized multi-center study in which 114 patients undergoing autologous hematopoietic stem cell transplantation were enrolled between October 2016 and October 2017. This study was approved by the institutional review board at the Dana Farber Harvard Cancer Center. Informed consent was obtained from all patients. This trial was registered at ClinicalTrials.gov (NCT02919670). Patients greater than 18-years-old diagnosed with MM or NHL and who were admitted for high-dose melphalan and ASCT were eligible for this trial. Patients who had chemotherapy or radiotherapy within 4 weeks of study entry or those with ongoing GI toxicities due to previous therapy were excluded. 

**Figure 1:**
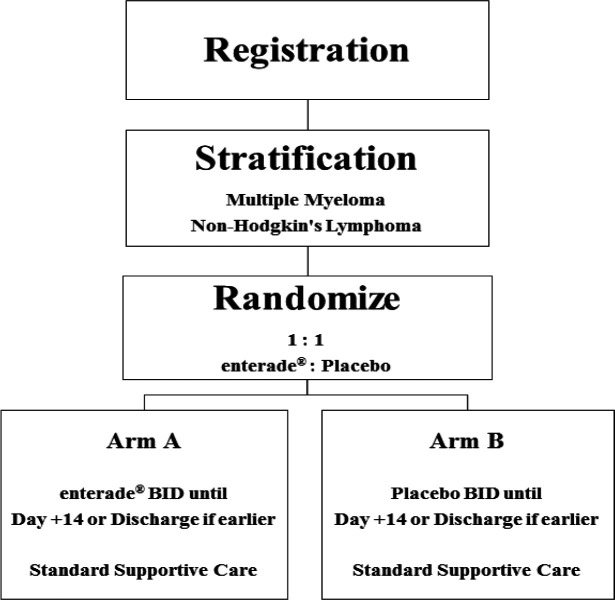
Study Design

Patients were randomized 1:1 to receive either two 8oz bottles of enterade^®^ or placebo starting on the day of admission through Day +14 (Figure 1), in addition to standard supportive measures. enterade^®^ is an amino acid-based electrolyte beverage, which consists of a combination of water, amino acids (valine, aspartic acid, serine, threonine, tyrosine), sodium chloride, natural flavor, sodium citrate, potassium chloride, gum acacia, calcium chloride, magnesium chloride, and sweetened with stevia leaf extract. The placebo consists of precisely the same ingredients excluding the amino acids. Both products were bottled identically and had the same color, taste and smell. 

Data on tolerability, compliance and GI toxicity was recorded from admission until day +14 after transplant. GI toxicity was scored by the CT-CAE 4.0 system from admission through Day + 14. Compliance with the study intervention was defined as consumption of 2 bottles of enterade^®^ or placebo daily for at least 11 days of the hospitalization. 

The initial primary analysis was an intent-to-treat analysis. An exploratory analysis of smaller compliant patient populations was also performed. Therefore, the analysis in this report includes all patients who consented (n = 114), many of whom consumed some amount of oral rehydration products (n = 99), and a cohort of patients who were fully compliant (n =13). Based on experience using standard of care as treatment, we anticipated grade 2 or higher diarrhea in 50% of patients in the placebo arm of the study and a reduction to 30% in the treatment arm. A sample size of 56 patients per arm provided 80% power to detect statistical significance (p < 0.05) between proportions using a one-tailed Fisher’s Exact Test. The Mann-Whitney U Test was used to compare descriptive data between cohorts.

**Table 1:** Baseline Characteristics (Demographics) (N=99)

	Arm					
	enterade (N=49)		Placebo (N=50)		All (N=99)	
	N	%	N	%	N	%
Age, median (range)	61 (25, 74)		59 (40, 77)		60 (25, 77)	
Sex						
Female	15	30.6	10	20	25	25.3
Male	34	69.4	40	80	74	74.7
Diagnosis						
MM	30	61.2	31	62	61	61.6
Newly Diagnosed	25		26		51	
Relapse	5		5		10	
NHL	19	38.8	19	38	38	38.4
Newly Diagnosed	7		9		16	
Relapse	12		10		22	
ECOG Performance Status						
0	17	34.7	20	40	37	37.4
1	30	61.2	29	58	59	59.6
2	2	4.1	1	2	3	3

**Table 2:** Incidence of Gastrointestinal Symptoms in the Intent-to-Treat Patient Cohort (n = 114).

Grade	enterade (n=58)		Placebo (n=56)
0-1	37 (63%)		28 (50%)
2	14 (24%)		15 (27%)
3	7 (12%)		12 (21%)
4	0		1 (2%)
0-1	19 (32%)	14 (25%)		
2	37 (63%)	42 (75%)		
3	2 (3%)	0 (0%)		
4	0 (0%)	0 (0%)		

## Results

Of the 114 patients enrolled, seven patients did not receive ASCT and one patient had a delayed transplant and thus no product was given. Five patients withdrew consent, one patient was removed from before starting intervention and one patient was removed due to an inability to swallow liquids. Thus, a total of 99 patients (74 male, 25 female) were included in this analysis. For patients consuming some amount of product, 49 were randomized to receive enterade^®^ and 50 placebo. Patient median age was 60 years (range, 25 - 77). MM was the diagnosis for the majority (62%) of the cohort. No statistical difference was observed in baseline characteristics between the enterade^®^ and placebo groups (Table 1). 

Overall compliance with the intended oral rehydration protocol was poor due to severe nausea (69%) and other GI toxicity symptoms such as oral mucositis, vomiting and dysphagia (28%). Of the 99 patients eligible for compliance analysis, none of the MM patients (0/61) met study compliance criteria (with oral rehydration - enterade^®^ or placebo) compared to 34% in the NHL group (13/38). Within the NHL patient group, 32% (n = 6) were compliant taking enterade^®^ versus 37% taking placebo (n = 7).

Table 2 shows the incidence and severity of a) diarrhea and b) nausea experienced by all patients in the intent-to-treat analysis. The total numbers of patients experiencing grade two or higher diarrhea was 36% for the enterade^®^ group (21/58) and 50% for the placebo group (28/56) (p = 0.097). Among patients who were able to be compliant with the planned treatment, only 1/6 (16%) of NHL patients taking enterade^®^ experienced ≥ grade 2 diarrhea versus 6/7 (86%) for patients with placebo (p = 0.025). The total number of patients experiencing grade 2 or higher nausea was 66% for the enterade^®^ group (39/58) and 75% for the placebo group (42/56) (p = 0.360).

## Discussion

The purpose of this study was to assess the tolerability and efficacy of enterade^®^ in reducing diarrhea in association with high-dose melphalan. We found that the administration of a twice daily oral supplement to patients receiving ASCT was not feasible due to a high incidence of gastrointestinal toxicities, regardless of assigned study intervention. Like others, we observed that the incidence of diarrhea was high for patients receiving high-dose chemotherapy and ASCT. Patients in the placebo cohort with ≥ grade 2 diarrhea was 50% as expected, while the incidence in the treatment cohort was slightly higher (36%) than anticipated (~30%). The difference between proportions was not significant (p = 0.097). However, amongst a small subset of subject who were able to maintain compliance with the study intervention, an exploratory analysis showed less severe diarrhea with enterade^®^ than placebo (p = 0.025). 

While we found that diarrhea was common in subjects regardless of assigned study intervention, an exploratory analysis suggested that enterade^®^ could provide benefit in reducing diarrhea when subjects are able to tolerate frequent and prolonged administration. However, the poor compliance observed with both enterade^®^ and placebo oral rehydration protocols following high-dose melphalan chemotherapy prevented a more meaningful evaluation of the study intervention on diarrhea. A high incidence of grade 2 or higher nausea was observed with ASCT in this study, which could potentially explain the lack of compliance and poor oral intake. While nausea is an established GI toxicity experienced during ASCT, we did not anticipate that it would have such a profound impact on the conduct of this study. Previous preclinical and clinical data had shown that the use of enterade^®^ was associated with lower rates of nausea and anti-emetic use (Yin et al., 2017; Luque et al., 2020). However, the rates of significant nausea in the current study with high-dose chemotherapy proved to be a main barrier to twice daily oral rehydration, especially when subjects are able to receive intravenous hydration as basic supportive care. As a consequence of limited oral intake due to nausea, well described nutritional problems such as malnutrition and dehydration are associated with high-dose chemotherapy and ASCT (Kewalramani et al., 2003). Additionally, patients also often experience significant changes and/or loss of taste with high-dose chemotherapy, causing even further difficulty with oral nutritional approaches (Ravasco, 2005). 

A major limitation of this study was the inability of more than 85% of patients to comply with drinking 2 bottles daily during ASCT due to high incidence of nausea secondary. To properly assess the benefits of enterade^®^ in diarrhea secondary to cancer or cancer therapy, future studies using this product should focus on study populations and/or cancer types with less upper GI toxicity. This might improve compliance and feasibility of a study to assess reductions in diarrhea. Based on the high degree of nausea, poor compliance, and the enterade^®^ mechanisms of action, it is possible that pre-dosing with enterade^®^ as a lead into treatment might provide additional practical and therapeutic value.

In conclusion, twice daily oral administration of enterade^®^ and placebo following high-dose chemotherapy and autologous stem cell transplantation was not feasible due to significant gastrointestinal toxicities. Within a small subset of subjects who were able to maintain compliance with the study intervention, an exploratory analysis showed significantly less severe diarrhea with enterade^®^ when compared to placebo and a trend toward the same in the larger ITT analysis. The use of enterade^®^ to prevent high dose chemotherapy associated diarrhea should be explored further in populations capable of reasonable oral intake.

*Disclosure Statement*

Laura Luque, Samuel N. Cheuvront and Katherine Mitchell are employees of Entrinsic Bioscience. All remaining authors report no conflict of interest.

*Data availability statement*

The data that supports the findings of this study is available from the corresponding author [LL], upon reasonable request. 
